# Examination of the mechanism of action of two pre-quit pharmacotherapies for smoking cessation

**DOI:** 10.1186/s12889-015-2596-2

**Published:** 2015-12-21

**Authors:** Stuart G. Ferguson, Julia A. E. Walters, Wenying Lu, Gudrun P. Wells, Natalie Schüz

**Affiliations:** School of Medicine, University of Tasmania, Private Bag 34, Hobart, TAS 7000 Australia; School of Health Sciences, University of Tasmania, Private Bag 135, Hobart, TAS 7001 Australia

**Keywords:** Nicotine patch, Varenicline, Pre-quit treatment, Smoking reduction

## Abstract

**Background:**

There is substantial scope for improvement in the current arsenal of smoking cessation methods and techniques: even when front-line cessation treatments are utilized, smokers are still more likely to fail than to succeed. Studies testing the incremental benefit of using nicotine patch for 1–4 weeks prior to quitting have shown pre-quit nicotine patch use produces a robust incremental improvement over standard post-quit patch treatment. The primary objective of the current study is to test the mechanism of action of two pre-quit smoking cessation medications—varenicline and nicotine patch—in order to learn how best to optimize these pre-quit treatments.

**Methods/Design:**

The study is a three group, randomized, open-label controlled clinical trial. Participants (n = 216 interested quitters) will be randomized to receive standard patch treatment (10 weeks of patch starting from a designated quit day), pre-quit patch treatment (two weeks of patch treatment prior to a quit day, followed by 10 weeks post-quit treatment) or varenicline (starting two weeks prior to quit day followed by 10 weeks post-quit). Participants will use study-specific modified smart-phones to monitor their smoking, withdrawal symptoms, craving, mood and social situations in near real-time over four weeks; two weeks prior to an assigned quit date and two weeks after this date. Smoking and abstinence will be assessed at regular study visits and biochemically verified.

**Discussion:**

Understanding how nicotine patches and varenicline influence abstinence may allow for better tailoring of these treatments to individual smokers.

**Trial registration:**

Australian New Zealand Clinical Trials Registry, ACTRN12614000329662 (Registered: 27 March 2014).

## Background

Cigarette smoking remains one of the leading causes of preventable death and disease worldwide [1, 2]. Perhaps not surprisingly, the majority of smokers indicate that they would like to quit [3]; despite this interest in quitting, however, the proportion of smokers who successfully quit each year is very low. Poor cessation rates can partly be attributed to the low uptake of efficacious smoking cessation methods [4]—a review [5] of unassisted (“cold turkey”) quit attempts concluded that the vast majority end in failure; however, even when smokers adopt effective cessation methods the most likely outcome is still failure. Even with the currently accepted gold standard treatment (i.e., pharmacotherapy combined with behavioural counselling) approximately 70 % [6, 7] of quit efforts fail. Thus, not only do interested quitters eschew current smoking cessation methods more often than not, but also when they are used they afford only modest improvements in the overall likelihood of quitting. Developing efficacious treatments that are accessible to a wide range of smokers will reduce tobacco-related deaths [8].

Numerous studies have been conducted over the last two decades aimed at developing new smoking cessation methods and treatments (e.g., [9, 10]), with some notable successes [11]. An alternate approach, however, is to optimize the use of existing smoking cessation methods and agents: improvements in quit rates can come from innovations in the way currently available treatments are used. Nicotine patch—a form of Nicotine Replacement Therapy (NRT)—is the most widely used pharmacotherapy to aid smoking cessation [4]. Patch treatment approximately doubles a smoker’s chance of maintaining longer-term abstinence (compared to placebo treatment) [7]. Nicotine patch was originally approved as an aid to abrupt cessation: smokers were instructed to stop smoking entirely and then start using the medication [7]. More recently, researchers have started to explore alternative methods of using patch, with an eye towards improving its effectiveness (e.g., [12, 13]). One alternative that has been of particular interest to researchers has been the incremental benefit of using nicotine patch for 1–4 weeks prior to quitting (so called nicotine “preloading” [14]), followed by the standard post-quit patch regime. Studies showed that nicotine patch preloading improves treatment efficacy: pre-quit nicotine patch use appears to produce a robust incremental improvement over standard patch treatment [14–16].

Why might using nicotine patch prior to quitting aid smoking cessation over and above the standard regime of starting patch on quit day? The most commonly voiced mechanism is that the non-contingently delivered nicotine from pre-quit nicotine patch treatment makes cigarettes less satisfying, thus blunting the reinforcement gained from smoking; this reduced satisfaction drives smoking reduction and subsequent cessation [14, 15]. Some support for this mechanism can be found in the nicotine patch literature. Previous studies have found that smokers rate cigarettes smoked while wearing a nicotine patch as less satisfying (e.g., [17–19]; c.f., [20]) and that the satisfaction obtained from smoking lapses predicts subsequent abstinence [20]. However, randomised studies that have examined pre-quit nicotine patch treatment’s effect on smoking satisfaction during the pre-quit period have found mixed results [15].

One reason for the mixed findings could be due to methodological shortcomings of the studies conducted to date. Studies of pre-quit nicotine patch use have typically relied on a participant’s retrospective recall to gather information on the effects of smoking while using patch. Such approaches are prone to systematic errors and bias [21–23]; these biases can mask legitimate trends in the data, and potentially lead to spurious conclusions.

More recently, our group conducted a small (*n* = 61) feasibility study designed to test the effect of pre-quit nicotine patch treatment on satisfaction gained from smoking. Using a single-group design [19], interested quitters monitored their smoking, affect and activities during two weeks of pre-quit nicotine patch treatment. In order to reduce the need for retrospective recall, data were collected in real-time using hand-held computers [22]. Participants were then followed-up after four weeks of post-quit treatment and abstinence was assessed. As expected, both smoking rate and satisfaction with smoking declined during the pre-quit nicotine patch period, and further analysis suggested that the degree of reduction achieved during the pre-quit period was related to the likelihood of abstinence at the four-week follow-up, although this relationship was not significant [19]. While these results are supportive of the posited mechanism of action, the absence of a control group meant that the study could not determine whether it was actually the pre-quit nicotine patch treatment that was driving the reductions in satisfaction and smoking rate observed during the pre-quit phase. The current study was designed to overcome this limitation by including a control group of participants who started nicotine patch treatment on quit day.

Nicotine patch is not the only smoking cessation pharmacotherapy to be used prior to quit: varenicline is also typically started 1–2 weeks prior to a quit attempt. Varenicline is the newest pharmacological smoking cessation agent and has been found to be more effective than other mono-therapies [6]. Interestingly, studies (e.g., [24]) have suggested that, like pre-quit nicotine patch treatment, the efficacy of varenicline may also, at least in part, be mediated via reductions in the satisfaction gained from smoking. Understanding how these two pre-quit agents influence abstinence may allow for better tailoring of treatment (e.g., if the proposed mechanism of action is confirmed, it may be beneficial to tailor the length of the pre-quit treatment phase until sufficient reduction has occurred, or to use reduction as an early indicator of treatment effectiveness [25]). Furthermore, understanding the effects of pre-quit treatment may provide targets for new medications.

The study will use a three-group, randomised, open-label study design. Participants will be randomized to one of two pre-quit treatment conditions, or a control group. Participants in the active pre-quit conditions will received two weeks of active medication treatment (nicotine patch or varenicline) prior to an assigned quit day, followed by up to 10 weeks of post-quit treatment; participants randomized to the control group will receive up to 10 weeks of nicotine patch treatment starting from their assigned quit day (so no treatment during the pre-quit phase). Using this design we plan to test four hypotheses: 1) Participants in the pre-quit treatment conditions will experience a significant reduction in the satisfaction gained from smoking over the two-week pre-quit period (compared to participants in the control group); 2) Participants in the pre-quit treatment conditions will experience a significant reduction in craving over the two-week pre-quit period (compared to participants in the control group); 3) Participants in the pre-quit treatment conditions will significantly reduce smoking rate over the two-week pre-quit period (compared to participants in the control group); and 4) Smoking reduction during the pre-quit phase will be positively associated with abstinence at four-week follow-up. A secondary objective of the study will be to look for moderators of treatment outcome (e.g., nicotine metabolism [26]) and to explore the effect of pre-quit treatment on lapses that occur post-quit.

## Methods

### Overview

Interested quitters will be monitored for two weeks before, and four weeks after, a quit attempt. Participants will be randomized to a Varenicline Group, a Pre-Quit Patch Group, or a Standard Patch Group. Participants randomized to the Standard Patch Group will receive nicotine patch treatment starting from their assigned quit day. Participants in the Pre-Quit Patch and Varenicline groups will receive two weeks of active medication treatment prior to an assigned quit day, followed by up to 10 weeks of post-quit medication. All participants will use a customized smart-phone [22] to monitor their smoking, affect, and activities in real-time for four weeks (two weeks prior to an assigned quit date and two weeks after this date). Abstinence will be assessed at regular study visits.

### Design

The study will use a three group, randomized, open-label controlled clinical trial study design.

### Participant recruitment

A community sample of interested quitters will be recruited from the greater Hobart (Tasmania, Australia) region using advertisements in local newspapers, flyers, and through targeted advertisements on social media websites such as Facebook [27].

### Inclusion/Exclusion criteria

To be eligible for enrolment, interested individuals will have to report: a) being ≥18 years old; b) smoking ≥10 cigarettes per day for the past three years; c) having a high motivation to quit smoking; d) be willing to use either patches or varenicline as part of a quit attempt; e) being able to read and write English; and f) be willing to consent to and complete the research tasks. Potential participants will be excluded if they: a) are currently enrolled in a smoking cessation trial, or have been within the past three months; or b) are unsuitable for treatment with either patches or varenicline (per current Australian prescribing guidelines [28]). Specifically, interested smokers will be excluded if they: Are a diabetic; have a mental illness or a history of repeated fits or convulsions (epilepsy); have kidney or liver problems; have a skin condition or disease such as allergic eczema or dermatitis, or allergies to any other medicines such as an itchy skin rash or swelling of the lips, face and throat; have uncontrolled, overactive thyroid gland; have a history of heart problems such as heart attack, chest pain or stroke, or untreated high blood pressure; or, are currently pregnant or breastfeeding. Initial screening will be conducted by phone and will be repeated in person by a trained study staff at the enrolment session. Screening will be overseen by a qualified general practitioner.

### Interventions

All participants will receive a standard paper-based self-help quitting booklet, provided prior to an assigned quit day. Participants randomized to either of the two pre-quit groups will receive medication for two weeks of active treatment (nicotine patches or varenicline) prior to their assigned quit day, followed by up to 10 weeks of post-quit treatment. Participants in the Standard Patch Group will start active patch treatment on the morning of their assigned quit day; they will not receive any medication during the pre-quit period. Medication (patches or varenicline) will be distributed at study visits (see below), with participants being provided enough medication to last until the next scheduled visit.

As all participants must report smoking ≥10 cigarettes per day to be eligible for enrolment, patch dose (for Pre-Quit Patch and Standard Patch participants) will be determined by baseline body weight per Australian guidelines [28]: participants who report a weight of ≥45 kg at enrolment will start on 21 mg/24-hr patches while participants who report a weight of <45 kg at enrolment will start on 14 mg/24-hr patches. Post-quit, participants will remain on the same dose for 10 weeks; dose will not be titrated downwards over the course of the study (again, in line with Australian guidelines). Participants in the Varenicline Group will receive 1 mg twice per day following a one-week titration period (0.5 mg once per day for three days, followed by 0.5 mg twice per day for four days). During each study visit participants will be asked about medication use since the last study visit (including frequency of use and adverse events) and any unused medication will be collected.

### Trial procedures

All participants will be asked to attend up to five study visits and to monitor their smoking using a modified smart-phone for a total of four weeks. Primary data collection will take place via the customized smart-phone (described below), with additional information obtained via questionnaires at study visits. All measures have been used and validated in the context of previous smoking studies.

At the beginning of the enrollment visit (Visit 1) eligible and interested smokers will be given a detailed overview of the study requirements and asked to provide written informed consent. Participants will then be randomized to their treatment group and assigned a quit date that will fall 17 days after initial enrolment. Participants will be instructed to quit smoking completely on their assigned quit day. During the initial visit participants will also provide two expired air carbon monoxide (CO) samples (see below), and a urine sample.

#### Baseline questionnaire assessment

During the initial visit, participants will be asked to complete a detailed smoking history and demographic characteristics questionnaire. Participants will be asked about basic demographic characteristics (e.g., age, gender, education, income, occupation, ethnicity, marital status etc.), current smoking and smoking history (e.g., age at initiation, cigarettes per day [CPD], years smoked, interest in quitting, past quit efforts etc.), nicotine dependence (as assessed by Fagerström Test for Nicotine Dependence [29] and the Nicotine Dependence Syndrome Scale [30]), alcohol use [31], feelings of depression [32], and satisfaction gained from cigarettes (using the modified Cigarette Evaluation Questionnaire [mCEQ]; [33]). As well as helping to characterize our sample, responses to these items will be used in our exploratory analyses of moderators of treatment outcome.

#### Ecological Momentary Assessment (EMA) data collection procedures

To collect near real-time data from participants as they undertake their quit effort, participants will monitor their smoking along with other study variables using modified smart-phones. At Visit 1 all participants will be provided with a smart-phone that has been stripped of its native functionality. These devices will be loaded with custom EMA data collection software designed by the study team. Participants will be asked to carry this device with them at all times for the following four weeks (until Visit 4). Participants will be asked to indicate, by pressing a button on the phone, each time they smoke a cigarette. The device will log the time and date of these events and store this data for later download and analysis.

During the pre-quit monitoring phase (i.e., Visit 1–3), the phone will be used to administer three types of assessments. Firstly, following 4–5 randomly selected cigarette reports per day, participants will be asked to complete an assessment of their current state (mood, withdrawal severity, craving etc.) as well as contextual and situational details (where the participants is, who they are with, what they are doing etc.). In addition to these “smoking assessments” the device is programmed to “beep” participants at a rate of approximately 4–5 times per day for randomly-selected non-smoking assessments (“random assessments”). During these assessments, participants will complete a series of items that parallels those administered during smoking assessments. Finally, participants will also complete end-of-day assessments to gather global reports on daily mood, craving and quitting self-efficacy. The two weeks of post-quit EMA monitoring will closely mirror that used in baseline monitoring, however post-quit assessments will include additional items pertaining to the lapse trigger(s) and the use (if any) of coping mechanisms [21]. Items in the proposed EMA assessments have been used and validated in previous EMA studies, and are reported in detail in resulting publications [21, 34]. Participants will be trained in EMA procedures and on assessment content before field monitoring commences formally (during Visit 1). Following procedures established in previous successful EMA studies [21], the second study visit (Visit 2) will be scheduled 2–4 days after participants start EMA field monitoring, at which time their data will be downloaded and checked, and they will receive further EMA training (if necessary). At the end of EMA monitoring (Visit 4) the study phones will be retrieved and re-used with subsequent participants.

#### Study visits 2–5

Participants will complete a further 4 study visits as part of the study. During each of these visits, CO and urine samples will be obtained for all participants, and satisfaction with smoking will be assessed using the mCEQ. During Visit 2 (14 days prior to quit day), participants in the Standard Patch group will be provided with one patch, which they will be instructed to apply on the morning of their assigned quit day. EMA data will be downloaded from all participants and checked for compliance, with extra training provided if necessary. Visit 3 will occur on a participants target quit day (17 days after enrollment). During this visit, in addition to the general tasks described above EMA data will be downloaded and checked for protocol compliance with additional training provided if necessary.

Study Visit 4 will occur 14 days after quit day and will mark the end of the EMA monitoring period and all phones will be handed back. The final study visit (Visit 5) will occur 28 days after participants target quit day. Participants who self-report 7-day point prevalent abstinence at this visit, verified by expired air CO (see below), will be provided a further six weeks of study medication. In addition to the general study visit tasks, during their final study visit participants will then complete an exit interview during which they will be asked about the study interventions and their experiences in the study. They will also be fully debriefed and offered the opportunity to ask any final questions about the study.

### Compensation and retention

In addition to receiving treatment, participants who complete the entire study will be paid $50. Participants will be paid for each visit that they complete, with the per visit payment escalating over the course of study visits ($10 at Visit 1, $20 at Visit 3, $20 at Visit 5). Once enrolled, participants will be sent a reminder e-mail and/or telephoned 24 h before a scheduled appointment. At the end of each session, the next session will be scheduled.

### Verification of smoking status

At each study visit participants will be asked about their current smoking status and any smoking that has occurred since the previous study visit (via a calendar-assisted 14-day timeline follow-back questionnaire). To verify self-report, expired air CO samples will be obtained at each study visit using a MICRO+ Smokerlyzer® (Bedfort Scientific, UK). Two samples will be recorded at each visit and if the average CO concentration of the two samples is <8 ppm, this will be taken as evidence of abstinence.

### Analytic plan

The proposed sample size (*n* = 216; 72 participants per group) was determined by the requirements of the primary research questions—namely, detecting reductions in satisfaction (H1) and craving (H2) scores, and smoking rate (H3), between the Standard Patch and the two pre-quit treatment groups at the end of the pre-quit treatment period (Visit 3). In a previous study [19] we observed moderate-to-large effects of pre-quit nicotine patch treatment on self-reported satisfaction with smoking, craving, and smoking rate during the pre-quit treatment period. Using these reductions as a proxy for between-group differences (assuming that these measures will not decrease during the pre-quit period for participants in the Standard Patch group), a conservative estimate of within-subject correlation of measures (0.5), our proposed sample will afford >90 % power to detect a difference in these measures. Our proposed sample size will also afford >80 % power to detect an effect of smoking reduction during the pre-quit phase on treatment outcomes (assessed at Visit 5; H4). Based on an earlier study [19] we expect to see ~6 % dropout before Visit 3 and as such we aim to recruit and enroll 229 participants in order to achieve our evaluable sample.

Our primary research questions involve reductions in satisfaction with smoking (H1), cigarette craving (H2), and smoking rate (H3) during the pre-quit treatment phase. These questions will be tested separately for each of the pre-quit groups using data gathered from the EMA field monitoring using mixed models growth curve analyses. The effect of smoking reduction on abstinence (H4) will be assessed in two ways. Firstly, reduction during the pre-quit phase will be used in a logistic regression model to predict biochemically verified 7-day point prevalent abstinence at Visit 5 (4 week abstinence). Treatment group will be included as a covariate. This outcome analysis will be conducted as intent-to-treat, with all participants who are randomized (Visit 2) being included in the analyses. Study dropouts will be counted as treatment failures. Next, as traditional intent-to-treat point prevalent abstinence analyses can obscure important aspects of the process of quitting smoking [13, 35], we will also use the real-time EMA data to examine time to first lapse using survival analyses.

In an exploratory analysis, we will compare the context and consequences of smoking lapses across the three treatment groups using data gathered during real-time assessments of smoking lapses [34]. Finally, we will examine whether individual differences in nicotine metabolism moderate treatment outcome. Nicotine metabolism will be expressed as the ratio of 3′-hydroxycotinine to cotinine in urine, as well as directly measuring their respective glucuronides (cotinine-N-glucuronide and 3′-hydroxycotinine-O-glucuronide). Instrumental analysis of urines will be undertaken using an Ultra Performance Liquid Chromatography-tandem mass spectrometry [36]. The main outcome measure will be total levels (free + conjugated) of cotinine and 3′-hydroxycotinine.

## Discussion

This study is designed to test the mechanism(s) though which pre-quit treatments—specifically nicotine patches and varenicline—aid cessation. Understanding how these two pre-quit treatments influence abstinence may allow for better tailoring of these efficacious treatments. Additionally, understanding the effects of pre-quit treatment with nicotine patch and varenicline may provide targets for new pharmacotherapies.

## Ethics and research governance

The project was reviewed and approved by the Tasmanian Health and Medical Research Ethics Committee (H0013619) prior to starting the recruitment process. Written informed consent will be obtained from all participants.
